# Resveratrol-Mediated Reversal of Doxorubicin-Induced Osteoclast Differentiation

**DOI:** 10.3390/ijms232315160

**Published:** 2022-12-02

**Authors:** Sunil Poudel, Gil Martins, M. Leonor Cancela, Paulo J. Gavaia

**Affiliations:** 1 Centre of Marine Sciences, University of Algarve, 8005-139 Faro, Portugal; 2 Faculty of Medicine and Biomedical Sciences (FMCB), University of Algarve, 8005-139 Faro, Portugal; 3 PhD Program in Biomedical Sciences, FMCB, University of Algarve, 8005-139 Faro, Portugal; 4 Algarve Biomedical Center, University of Algarve, 8005-139 Faro, Portugal

**Keywords:** osteoclast differentiation, oxidative stress, resveratrol, MitoTEMPO, doxorubicin, secondary osteoporosis

## Abstract

Secondary osteoporosis has been associated with cancer patients undertaking Doxorubicin (DOX) chemotherapy. However, the molecular mechanisms behind DOX-induced bone loss have not been elucidated. Molecules that can protect against the adverse effects of DOX are still a challenge in chemotherapeutic treatments. We investigated the effect and mechanism of DOX in osteoclast differentiation and used the *Sirt 1* activator resveratrol (RES) to counteract DOX-induced effects. RAW 264.7 cells were differentiated into osteoclasts under cotreatment with DOX and RES, alone or combined. RES treatment inhibited DOX-induced osteoclast differentiation, reduced the expression of osteoclast fusion marker *Oc-stamp* and osteoclast differentiation markers *Rank*, *Trap*, *Ctsk* and *Nfatc1*. Conversely, RES induced the upregulation of antioxidant genes *Sod 1* and *Nrf 2* while DOX significantly reduced the *FoxM1* expression, resulting in oxidative stress. Treatment with the antioxidant MitoTEMPO did not influence DOX-induced osteoclast differentiation. DOX-induced osteoclastogenesis was studied using the *cathepsin*-K zebrafish reporter line (*Tg[ctsk:DsRed]*). DOX significantly increased *ctsk* signal, while RES cotreatment resulted in a significant reduction in *ctsk* positive cells. RES significantly rescued DOX-induced mucositis in this model. Additionally, DOX-exposed zebrafish displayed altered locomotor behavior and locomotory patterns, while RES significantly reversed these effects. Our research shows that RES prevents DOX-induced osteoclast fusion and activation in vitro and in vivo and reduces DOX-induced mucositis, while improving locomotion parameters.

## 1. Introduction

Doxorubicin (DOX), a first-line chemotherapeutic agent, is known for its cytotoxic effects, characterized by the accumulation of reactive oxygen species (ROS) and reactive nitrogen species (NOS) [1]. DOX has been shown to increase systematic bone loss and reduce osteoblast differentiation [2,3,4]. Bone homeostasis depends on the cross-talk between bone-forming by osteoblasts and bone resorption by osteoclasts. Any imbalance of this coupled process results in diseases such as osteopenia and osteoporosis. The presence of ROS can result in increased reduction–oxidation reactions and cause interruption of normal biological functions, leading to oxidative stress and disrupting bone homeostasis [5]. Cytokine receptors such as TNF-α, IL-1, TGF-β and G protein-coupled receptors have been shown to generate ROS, that will serve as a mediator for the activation of downstream signaling pathways [6]. This receptor-mediated generation of ROS plays a crucial role in RANKL-RANK-induced osteoclastogenesis [7]. ROS produced by macrophages plays a critical role in cellular defense as well as in receptor-mediated pathways such as PI3K, NF-kB and AKT [6,8]. During osteoclast differentiation, ROS acts as a secondary messenger in RANKL-RANK dependent signaling pathways such as TRAF, NFATC1, AKT, and MAPKS [7,9,10,11]. Similarly, the NADPH oxidase system (Nox) plays a major role in ROS-mediated bone resorption; however, the knockdown mouse model of Nox did not show any reduction in ROS or any bone abnormalities [12,13]. ROS produced at various subcellular sites of macrophages, such as the mitochondria electron transfer chain, are responsible for the differentiation of osteoclasts [14]. Likewise, NFATC1, a master regulator for osteoclast differentiation, could be a crucial downstream modulator of RANKL-mediated ROS signaling [15]. 

FoxM1 is a member of the forkhead box transcription factor family, like the FoxO transcription factors. However, in contrast to those, which are activated in quiescent cells and inhibit proliferation, FoxM1 is only expressed in proliferating cells and has a critical role in cell-cycle progression [16,17,18]. Chemotherapeutic agents such as DOX, epirubicin, paclitaxel, lapatinib, gefitinib, imatinib and cisplatin have been reported to exert their cytotoxic and cytostatic capacities through FoxO3 and FoxM1 [19]. The expression of FoxM1 is induced by increased oncogenic stress requiring ROS, which upregulates FoxM1 in a negative feedback loop. This counteracts the elevated intracellular ROS levels by stimulating the expression of antioxidant enzyme genes to protect dividing cells and tumor cells from oxidative stress [16]. In addition, FoxM1 has been shown to be a downstream target of p38 MAPK, which is also downregulated in response to DOX and epirubicin exposure [20,21].

Sirt1 inhibits bone resorption by inhibiting NF-kB signaling in osteoclast lineage cells [22,23,24] and promotes bone formation via the deacetylation of FoxOs [25]. The activation of Sirt1 with the natural polyphenol resveratrol (RES) has been proven to attenuate the loss of bone mass caused by ovariectomy [22,23], hind limb unloading [24,26], or ageing in mice [27]. Additionally, RES also inhibits osteoclast formation in vitro through the inhibition of ROS [28,29,30], increases catalase and superoxide dismutase (SOD) activity [31] and protects mitochondria against oxidative stress [32].

Similarly, the mitochondrial superoxidase scavenger, (2-(2,2,6,6-Tetramethylpiperidin-1-oxyl-4-ylamino)-2-oxoethyl) triphenylphosphonium chloride (MitoTEMPO, MT) protects against mitochondrial dysfunction by scavenging ROS [33,34]. N-acetyl cysteine, an antioxidant widely used as a nutritional supplement, and free radical scavenging enzymes such as superoxide dismutase and diphenyleneiodonium were shown to inhibit osteoclast formation [35,36]. Mitochondrial ROS (mtROS) are essential for the hypoxic enhancement of osteoclast differentiation [37]. However, on osteoclasts, pyruvate dehydrogenase is not affected during hypoxia, which facilitates the entry of pyruvate to a TCA cycle and allows mitochondrial metabolic flux, a continuation of hypoxic oxidative phosphorylation results in the accumulation of mitochondrial ROS in osteoclasts [37,38]. Mitochondrial antioxidants have been shown to reverse the effect of mtROS on osteoclast differentiation [14,36], as was reported for mitochondria-specific antioxidant MitoQ, which was shown to prevent the hypoxic induction of NF-kB, NFAT pathway, CREB and HIF signaling molecules during osteoclast differentiation and suppressed the RANKL-induced differentiation of RAW264.7 cells into multinucleated and TRAP-positive osteoclasts [14]. Tafazzin is a mitochondrial enzyme crucial for cardiolipin remodeling. Knocking down tafazzin in cardiac myocytes decreased the levels of cardiolipin and increased mtROS resulting in mitochondrial dysfunction. MT treatment normalized tafazzin knockdown-induced mtROS production and counteracted the resultant apoptosis [39].

DOX has also been shown to increase bone loss by restraining osteoblastogenesis [40] and increasing osteoclastogenesis [41]. In addition, DOX and epirubicin exposure were shown to downregulate FoxM1 [20,21], leading to the activation of osteoclast differentiation [42]. We hypothesized that the involvement of FoxM1 on DOX-induced osteoclast differentiation is a factor contributing to DOX-induced bone loss. Therefore, in this study, we investigated the involvement of FoxM1 on DOX-induced osteoclast differentiation and examined the potential of antioxidants to reverse these negative effects of DOX on bone. In addition, we used a *cathepsin*-K reporter zebrafish transgenic line (*Tg[ctsk:DsRed*]) [43] to investigate the effects of DOX, RES and MT on the activation of osteoclasts and to investigate the capacity of antioxidants to counteract the effect of DOX in vivo. 

## 2. Results

### 2.1. Cytotoxic Effect of Resveratrol, Doxorubicin and MitoTEMPO

**Figure 1:**
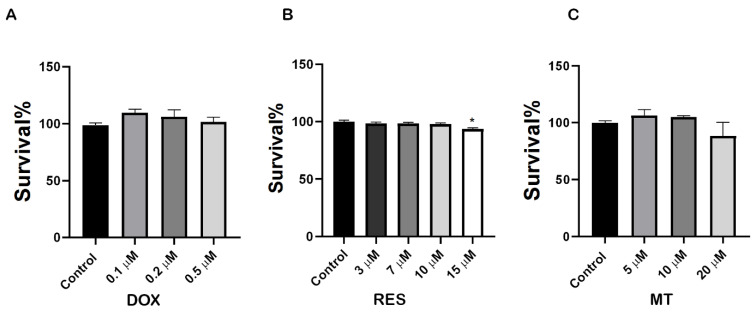
Cytotoxicity of doxorubicin and antioxidants on RAW 264.7 cells. RAW 264.7 cells were exposed to different concentrations of Doxorubicin (DOX) (**A**), Resveratrol (RES) (**B**) and MitoTEMPO (MT) (**C**) and an XTT assay was performed. Cells were treated for 3 days with RES and MT, and for 3 h with DOX. One-way ANOVA, Tukey’s multiple comparisons test, *—*p* ≤ 0.05.

To investigate the molecular mechanisms underlying the effect of RES, MT and DOX on osteoclast differentiation, we first performed an XTT assay to analyze the potential cytotoxic concentration of these compounds on RAW 264.7 cells. As shown in Figure 1A, DOX exposure at a concentration range of 0.1 µM–0.5 µM did not show any toxicity. All concentrations tested, up to 10 µM RES, did not produce any toxic effect on the cells after 4 days of treatment (Figure 1B). MT did not show any toxic effect on cells after 4 days of treatment at concentrations from 5 µM to 20 µM (Figure 1C). Therefore, 10 µM RES, 10 µM MT and 0.1 µM DOX (3 h exposure with 72 h intervals) were determined as non-toxic and selected for further experiments with RAW 264.7 cells.

### 2.2. Inhibition of Doxorubicin-Induced Osteoclastogenesis by RES

**Figure 2:**
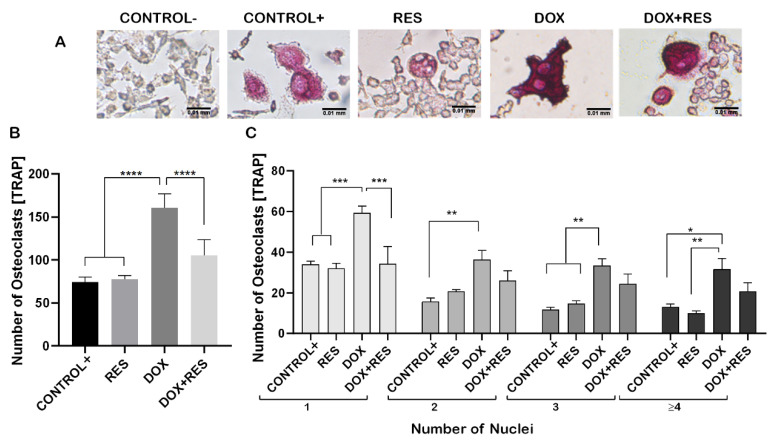
Resveratrol prevents doxorubicin-induced osteoclast differentiation. RAW 264.7 cells were cultured for 4 days with M-CSF (30 ng/mL) and RANKL (50 ng/mL) and treated with resveratrol (RES) and doxorubicin (DOX) alone or combined. TRAP-positive osteoclastic cells (**A**). Number of TRAP-positive cells (**B**). Number nuclei in TRAP-positive cells from the different treatments. Cells were grouped according to the number of nuclei: 1, 2, 3 or more than 4 (≥4) (**C**). One-way ANOVA, Tukey’s multiple comparisons test, *—*p* ≤ 0.05, **—*p* ≤ 0.01, ***—*p* ≤ 0.001, ****—*p* ≤ 0.0001.

Raw 264.7 cells were differentiated into the osteoclastic lineage by induction with M-CSF (30 ng/mL) and RANKL (50 ng/mL) in co-treatment with DOX and RES to verify the effect of exposure to these molecules, alone or in combination, over osteoclastogenesis (Figure 2A). DOX treatment induced a significant increase in the number of tartrate-resistant acid phosphatase (TRAP)-positive cells compared to a positive control (with M-CSF and RANKL). However, while co-treating with DOX and RES simultaneously, the number of TRAP-positive osteoclastic cells was significantly reduced compared to DOX alone (Figure 2B). In addition, the number of multinucleated osteoclasts was significantly increased in the DOX treatment compared to the RES and control groups (Figure 2C).

### 2.3. Resveratrol Inhibits Doxorubicin-Induced Osteoclast Differentiation Marker Genes

**Figure 3:**
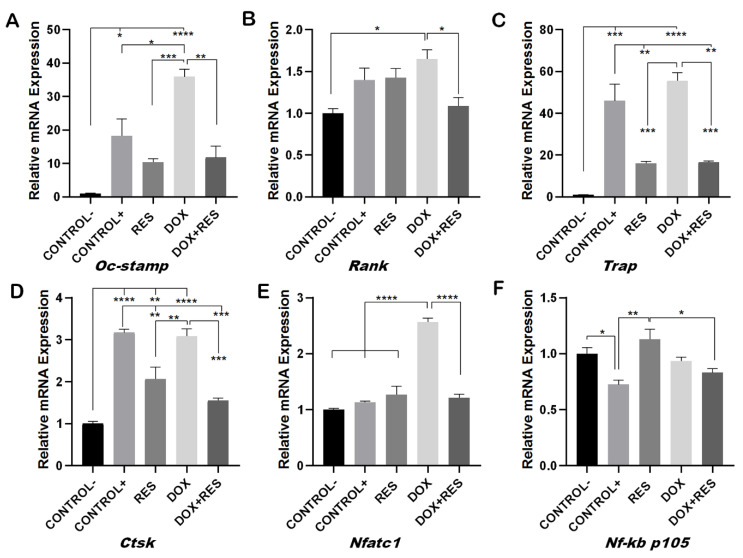
Inhibition of doxorubicin-induced osteoclast differentiation by resveratrol. RAW 264.7 cells were cultured for 4 days with M-CSF (30 ng/mL) and RANKL (50 ng/mL) and treated with resveratrol (RES) and doxorubicin (DOX) alone or combined. Levels of mRNA expression for *Oc-stamp* (**A**), *Rank* (**B**) *Trap* (**C**), *Ctsk* (**D**), *Nfatc1* (**E**) and *Nf-kb p105* (**F**) were analyzed by qPCR. One-way ANOVA, Tukey’s multiple comparisons test, *—*p* ≤ 0.05, **—*p* ≤ 0.01, ***—*p* ≤ 0.001, ****—*p* ≤ 0.0001.

During osteoclast differentiation OC-STAMP, *RANK, TRAP, CTSK*, *NF-KB* and *NFATC1* play essential roles. The mRNA expression of these differentiation markers of osteoclastogenesis was examined after 96 h of differentiation in Raw 264.7 cells. DOX treatment showed increased numbers of multinucleated osteoclasts whereas, when combined with RES, the number of multinucleated osteoclasts decreased (Figure 2). Therefore, we hypothesized that DOX increases the fusion of the osteoclast. As expected, the mRNA levels of the osteoclast fusion marker osteoclast stimulatory transmembrane protein (*Oc-stamp*) were significantly increased on DOX treatment, while in combination with RES, the *Oc-stamp* was significantly downregulated (Figure 3A). Similarly, DOX treatment significantly increased *Rank* expression compared to the negative control; however, upon co-treatment with RES, *Rank* expression was significantly reduced compared to DOX (Figure 3B). Similarly, *Trap* expression was reduced upon RES treatment as compared to the positive control and upon combined treatment, and *Trap* mRNA expression was significantly reduced by RES as compared to DOX alone (Figure 3C). *Ctsk* mRNA expression was significantly increased upon DOX treatment as compared to other groups; however, RES significantly decreased the DOX-induced expression of *Ctsk (*Figure 3D).

Furthermore, RES significantly reduced the expression of *Nfatc1* as compared to the positive control whereas, when combining RES with DOX, RES significantly reduced the *Nfatc1* expression as compared to DOX alone (Figure 3E). *Nf-kb p105* was significantly upregulated upon RES treatment, whereas when combining DOX with RES, the expression of *Nf-kb p105* was significantly reduced as compared to RES alone (Figure 3F). This indicates that RES inhibits *Nf-kb*-induced osteoclast differentiation. 

### 2.4. Involvement of FoxM1 on Osteoclast Differentiation and Oxidative Stress

**Figure 4:**
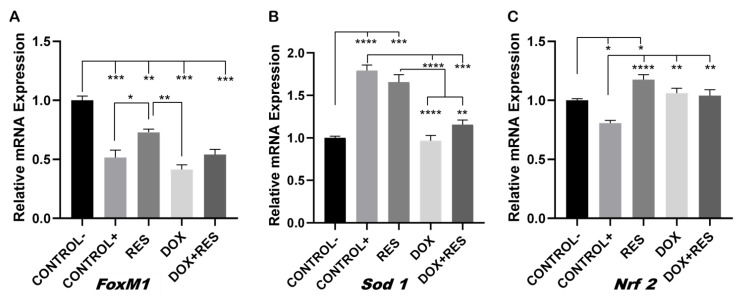
Involvement of FoxM1 on osteoclast differentiation and oxidative stress. RAW 264.7 cells were cultured for 4 days with M-CSF (30 ng/mL) and RANKL (50 ng/mL) and treated with resveratrol (RES), MitoTEMPO (MT) and doxorubicin (DOX) alone or together. RNA from the cells was obtained, and expression levels for *FoxM1* (**A**), *Sod 1* (**B**) and *Nrf 2* (**C**) were analyzed by qPCR. One-way ANOVA, Tukey’s multiple comparisons test, *—*p* ≤ 0.05, **—*p* ≤ 0.01, ***—*p* ≤ 0.001, ****—*p* ≤ 0.0001.

*FoxM1* transcription factor controls cell proliferation by promoting cell cycle progression and is a critical regulator of oxidative stress. Elevated *FoxM1* levels downregulate ROS by stimulating the expression of ROS scavenger genes such as *Sod 1*, *Nrf 2,* among others [16,44,45]. The expression of *FoxM1* was significantly reduced on osteoclasts (positive control) as compared to the undifferentiated cells (negative control), suggesting the strong involvement of ROS in the differentiation processes (Figure 4A). 

The expression of *FoxM1* was significantly upregulated on the RES group relative to the positive control and DOX, whereas in the group co-treated with DOX and RES, the expression of *FoxM1* only showed a slight increase which was not significantly different from the DOX or the other groups (Figure 4A). The expression of antioxidant gene *Sod 1* was significantly reduced in the DOX group as compared to RES-treated cells and positive control; however, the co-treatment group showed a small rescue of Sod 1 expression (Figure 4B). RES also significantly upregulated *Nrf 2* mRNA expression as compared to the positive and negative controls. However, DOX and DOX + RES also upregulated *Nrf 2* mRNA as compared to the positive control (Figure 4C).

### 2.5. Effect of Mitochondrial Antioxidant on Doxorubicin-Induced Osteoclast Differentiation

**Figure 5:**
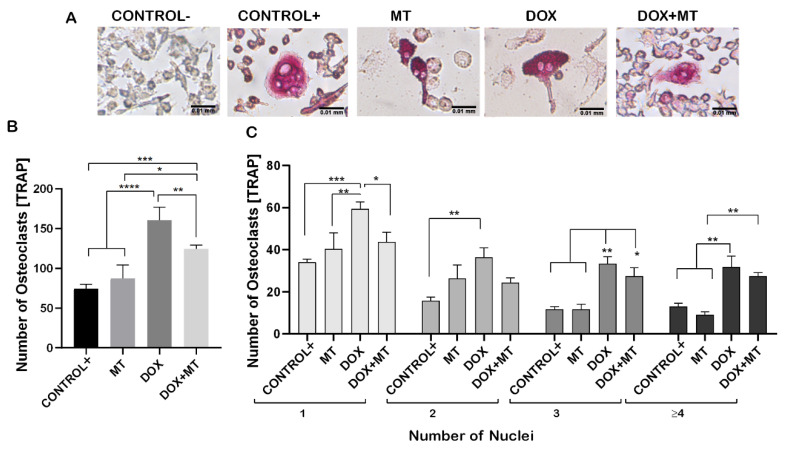
Effect of MitoTEMPO on doxorubicin-induced osteoclast differentiation. RAW 264.7 cells were cultured for 4 days with M-CSF (30 ng/mL) and RANKL (50 ng/mL) and treated with MitoTEMPO (MT) and Doxorubicin (DOX) alone or together. The cells were stained for TRAP (**A**). The number of TRAP-positive cells (**B**). Cells were grouped according to number of nuclei: 1, 2, 3 or more than 4 (≥4) (**C**). One-way ANOVA, Tukey’s multiple comparisons test, *—*p* ≤ 0.05, **—*p* ≤ 0.01, ***—*p* ≤ 0.001, ****—*p* ≤ 0.0001.

To investigate the involvement of ROS during osteoclast differentiation, we used MitoTEMPO (MT, triphenylphosphonium chloride), a mitochondrial superoxide scavenger, which blocks mitochondrial ROS and intracellular ROS generation [46,47]. MT was shown to significantly reduce the DOX-induced osteoclast differentiation, as observed by the TRAP-positive cells (Figure 5A,B). No significant difference was observed in the number of TRAP-positive cells on MT treatment compared to positive control. While, when combining DOX with MT, the number of single nucleated osteoclasts was significantly lower on the combined treatment with DOX and MT compared to DOX alone. However, the number of multinucleated osteoclasts (with three or more nuclei) was not significantly different on DOX compared to DOX and MT (Figure 5C). The number of multinucleated TRAP positive cells (with 3 or 3+ nuclei) were significantly increased on the combined treatment with DOX and MT as compared to MT alone and control (Figure 5C). Therefore, MT showed a limited effect in counteracting the DOX-induced osteoclast differentiation. 

### 2.6. MitoTEMPO Is Unable to Reverse Doxorubicin-Induced Osteoclast Markers Genes

**Figure 6:**
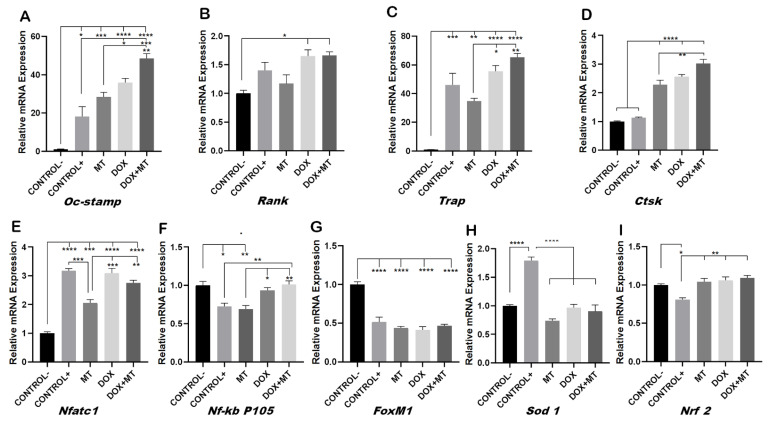
Effect of MitoTEMPO on doxorubicin-induced osteoclast differentiation and oxidative stress markers. RAW264.7 cells were cultured for 4 days with M-CSF (30 ng/mL) and RANKL (50 ng/mL) and treated with MitoTEMPO (MT) and Doxorubicin (DOX) alone or together. Levels of expression for *Oc-stamp* (**A**), *Rank* (**B**), *Trap* (**C**), *Ctsk* (**D**), *Nfatc1* (**E**), *Nf-kb p105* (**F**), *FoxM1* (**G**), *Sod1* (**H**) and *Nrf 2* (**I**) were analyzed by qPCR. One-way ANOVA, Tukey’s multiple comparisons test, *—*p* ≤ 0.05, **—*p* ≤ 0.01, ***—*p* ≤ 0.001, ****—*p* ≤ 0.0001.

To investigate whether MT was able to reverse the DOX-induced osteoclast differentiation, we investigated the osteoclast fusion and differentiation markers on MT and DOX alone and in combination. The DOX-induced expression of the osteoclast fusion marker *Oc-stamp* was not reduced when combined with MT (Figure 6A). The DOX-induced expression of differentiation markers of osteoclastogenesis *Rank, Trap, Ctsk* and *Nfatc1* were unaffected when combined with MT (Figure 6B–E). *Trap* and *Nfatc1* were significantly reduced upon MT treatment as compared to the positive control (Figure 6C,E). The DOX-induced expression of *Nf-kb p105* was also not affected by MT cotreatment (Figure 6F). The mRNA expression of *FoxM1* was significantly reduced on all treatment groups as compared to the negative control (Figure 6G). Since MT, a mitochondrial superoxide scavenger, blocks mitochondrial ROS and intracellular ROS generation [46,47], the mRNA expression of *Sod 1* was significantly lower as compared to the positive control. However, the DOX and DOX + MT treatments also showed a downregulation of *Sod1* (Figure 6H). *Nrf 2* mRNA expression was upregulated in all treatments as compared to the positive control. However, it showed no significant differences between MT and DOX alone or in combination (Figure 6I).

### 2.7. In Vivo Reversal of Doxorubicin-Induced Osteoclast Differentiation by Resveratrol

**Figure 7:**
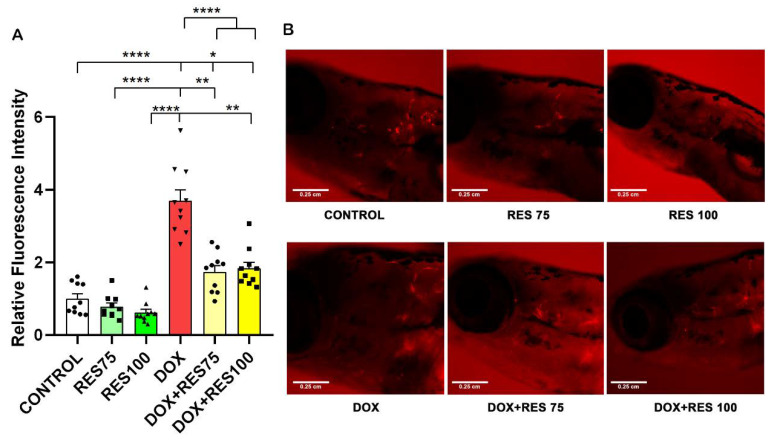
Inhibitory effect of resveratrol on doxorubicin-induced stimulation of osteoclasts. The *ctsk* reporter zebrafish transgenic line (*Tg[ctsk:DsRed*]) at 25 dpf was exposed to different concentrations of resveratrol (RES, 75 µM and 100 µM) alone or together with Doxorubicin (DOX, 17.2 µM) for 96 h and *ctsk* signal was measured in the head area. Quantification of fluorescence intensity between the treatment groups performed using fluorescent images acquired with the same parameters and analyzed with ImageJ (**A**), and *ctsk*-positive cells in the head of fish from the different treatment groups (**B**). One-way ANOVA, Tukey’s multiple comparisons test, *— *p* ≤ 0.05, **—*p* ≤ 0.01, ****—*p* ≤ 0.0001.

We investigated the effect of DOX and RES, alone or in combination, in a *ctsk* reporter zebrafish transgenic line (*Tg[ctsk:DsRed*]) [43]. At 25 dpf, the zebrafish were exposed to DOX (17.2 µM) alone, RES (75 µM and 100 µM) alone or in combination for 96 h. After 96 h, *ctsk* positive cells in the head area were identified by fluorescence microscopy; *ctsk* positive cells were significantly higher in DOX-treated groups (Figure 7A,B). While when combining DOX with RES, the number of *ctsk* positive cells was significantly reduced as compared to DOX alone (Figure 7B), suggesting that the RES significantly prevented DOX-induced osteoclast differentiation in vivo. 

### 2.8. In Vivo Reversal of Doxorubicin Induced Mucositis by Resveratrol

**Figure 8:**
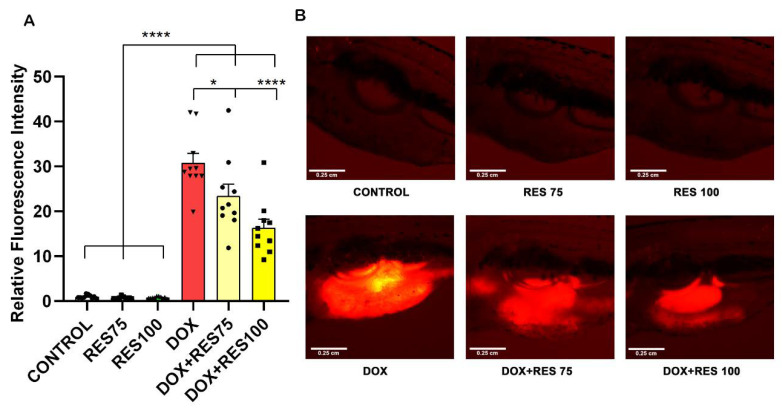
Reversal of Doxorubicin-induced mucositis by resveratrol. The *ctsk* reporter zebrafish transgenic line (*Tg[ctsk:DsRed*]) was exposed to different concentrations of resveratrol (RES, 75 µM and 100 µM) alone or together with doxorubicin (DOX, 17.2 µM) for 96 h. The quantification of fluorescence intensity between the treatment groups performed using fluorescent images acquired with the same parameters and analyzed with ImageJ (**A**). *ctsk* signal in the abdominal area of fish from the different treatment groups (**B**). One-way ANOVA, Tukey’s multiple comparisons test, *—*p* ≤ 0.05, ****—*p* ≤ 0.0001.

Our results reveal that DOX increased mucositis on zebrafish. The expression of *ctsk* on the abdominal region was extremely high in DOX-treated groups compared to control and RES groups (Figure 8A,B). While when combining DOX with 75 µM or 100 µM RES, the mucositis was significantly reversed, as shown by the reduction in fluorescence signal on the abdominal region (Figure 8B).

### 2.9. MitoTEMPO Is Unable to Reverse Doxorubicin-Induced Osteoclast Differentiation In Vivo

**Figure 9:**
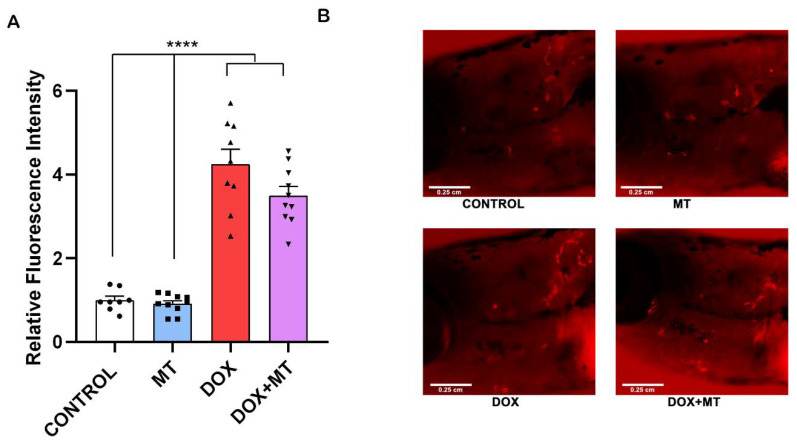
Effect of MitoTEMPO on doxorubicin-induced stimulation of *ctsk* cells. The *ctsk* reporter zebrafish transgenic line (*Tg[ctsk:DsRed*]) at 25 dpf was exposed to MitoTEMPO (MT, 20 µM) alone or together with doxorubicin (DOX, 17.2 µM) for 96 h. Quantification of fluorescence intensity between the treatment groups (**A**) and the *ctsk* signal in the head area of fish from the different treatment groups (**B**). One-way ANOVA, Tukey’s multiple comparisons test, ****—*p* ≤ 0.0001.

We investigated the effect of DOX and MT, alone or in combination, in a *ctsk* reporter zebrafish transgenic line (*Tg[ctsk:DsRed*]). Our results show that, when combining MT with DOX, the *ctsk* positive cell’s fluorescence signal was not significantly altered compared to the DOX treatment group (Figure 9A,B). Therefore, MT at 20 µM concentration was unable to reverse the DOX-induced osteoclast differentiation as indicated by *ctsk* positive cells. 

### 2.10. Doxorubicin Decreases Locomotor Activity of Zebrafish

**Figure 10:**
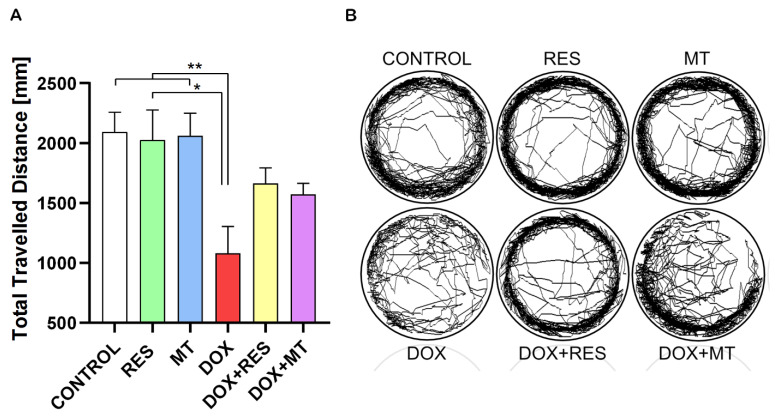
Effect of doxorubicin on locomotor activity of zebrafish post-larvae. *Ctsk* reporter zebrafish transgenic line (*Tg(ctsk:DsRed*)) were exposed to different concentrations of resveratrol (RES), MitoTEMPO (MT) alone or together with doxorubicin (DOX) for 24 h. Locomotor activity was measured on Zantiks MWP system. Total distance travelled with RES (100 µM), MT (20 µM) and DOX (17.2 µM) alone or together (**A**). The locomotory pattern of fish between the treatment groups (**B**). One-way ANOVA, Tukey’s multiple comparisons test, *—*p* ≤ 0.05, **—*p* ≤ 0.01.

Zebrafish at 25 dpf were exposed to RES (100 µM), MT (20 µM) and DOX (17.2 µM) alone or in different combinations for 24 h, and the locomotor activity was analyzed using a Zantiks MWP. Figure 10A show the total travelled distance of the post-larvae treated with RES, MT and DOX. The travelled distance was significantly reduced by DOX compared to the control treatment and to both antioxidants alone (RES and MT). The locomotion pattern of these groups is shown in Figure 10B. In the combination treatments with DOX and RES or DOX and MT, the distance travelled did not show any significant differences between DOX alone or in combination. However, in the fish under co-treatment with RES and DOX, there was an improvement on locomotive pattern as compared to DOX. 

## 3. Discussion

Doxorubicin (DOX) is a chemotherapy agent that increases reactive oxygen species (ROS) and is known to decrease *FoxM1* expression, which is required for the proliferation of cells [1]. FoxO3 and FoxM1 function downstream of PI3K-Akt, whilst Ras-ERK and JNK/p38MAPK signaling pathways are crucial for cell survival, proliferation, differentiation, and cell cycle control. This chemotherapeutic agent influences cellular toxicity through FOXO3 and FoxM1 signaling, affecting cell survival, proliferation, differentiation via cell cycle control resulting in cell termination by apoptosis or senescence [19]. Patients under DOX treatment showed increased systemic bone loss during chemotherapy [2], and a significant depletion of bone mass was observed upon DOX exposure in rats [3,4]. This depletion of bone mass was established to be caused by an imbalance between osteoblast and osteoclast activity. Here, we showed that osteoclast activity was significantly increased upon DOX exposure, as shown by increased numbers of TRAP-positive cells. The number of multinucleated osteoclasts was significantly higher on the DOX treatment, which is suggestive evidence of increased resorption resulting on systematic bone loss during chemotherapy [2]. However, co-treatment with DOX and RES decreased the TRAP-positive cells and osteoclast markers. *Oc-stamp* encodes for a transmembrane protein required for the fusion of osteoclasts [48] that was highly expressed on DOX-treated RAW 264.7 cells’ differentiation, suggesting that DOX promoted an increased fusion of the macrophage/monocyte lineage during osteoclastogenesis, forming larger, and therefore, more active osteoclasts. Interestingly, when combining DOX with RES, there was a significant reduction in *Oc-stamp* mRNA expression, suggesting that RES prevents the fusion of macrophages. 

Resveratrol (RES) is known to influence a broad range of signaling pathways including sirtuins, kinases, steroid receptors, lipo- and cyclooxygenases [31,45]. Sirt1 signaling is an important mediator of stress resistance and antiaging effects [49]. By activating Sirt1, RES epigenetically modifies the expression of mesenchymal stem cells, favoring osteoblast differentiation and decreasing adipocyte formation. Similarly, the RES-mediated apoptosis of osteosarcoma cells involves Sirt1 activation. RES induced apoptosis in osteosarcoma cells in a dose-dependent manner but did not significantly affect normal osteoblasts [47,48]. Gehm et al. [50] showed that RES functions as an agonist for estrogen receptor-mediated transcription due to its similar structure to diethylstilbestrol, a synthetic estrogen [50]. Similarly, β-estradiol dose-dependently decreased M-CSF and RANKL-induced osteoclast differentiation in myelomonocytic precursors [51]. The inhibition of osteoclast differentiation by RES might be due to its similar structure to estrogen [50]. RelA/p65 is responsible for the NF-κB heterodimer formation, nuclear translocation and signal activation. RES has been shown to inhibit RANKL-induced osteoclast differentiation by the deacetylation of RelA/p65 at the lysine 310 subunit of NF-κB [52,53]. Similarly, *Nf-kb p105* served as *Nf-κb* precursors and inhibitors of NF-κB dimers [52]. In this study, RES significantly increased *Nf-kb p105,* suggesting that RES inhibits NF-kB dimer formation, thus inhibiting nuclear translocation and subsequent inhibiting NF-kB signaling. Similarly, receptor-mediated ROS generation plays a crucial role in RANKL-induced osteoclast differentiation. RANKL stimulation increases ROS in pre-osteoclasts via NADPH oxidase or increased mitochondrial ROS [9,14,54]. Taken together, this study provides evidence of an increased DOX-induced osteoclast differentiation and fusion and shows the ability of RES to counteract DOX-induced osteoclast activation. Previously, DOX was shown to be able to activate the immune system with an increase in inflammatory cytokines (IL-1β, IL-6 and TNFα) [55]. The osteoclast differentiation markers *Rank, Trap, Ctsk* and *Nfatc1* were significantly upregulated by DOX exposure, confirming the results observed in the DOX treatment regimen, with a significant increase in osteoclast differentiation. On the other hand, RES treatment was shown to decrease the expression of an osteoclast fusion marker (*Oc-stamp*) and differentiation markers (*Trap* and *Ctsk*) compared to the positive control with M-csf and Rankl induction. It has been shown that the inhibition of *Oc-stamp* completely inhibits the fusion and multinucleation of osteoclasts, resulting in reduced resorption [56]. These results are in line with the experimental data presented in our work, with a reduction in the multinucleated osteoclasts and a downregulation of differentiation markers upon treatment with RES, as observed both in vivo and in vitro. Taken together, our results show that RES significantly reduces DOX-induced osteoclast fusion and differentiation. Therefore, it is possible to suggest that a combined therapy of DOX with the supplementation of RES would significantly help in preventing DOX-induced bone resorption. 

In our study, DOX exposure significantly reduced *FoxM1* expression during the osteoclastic differentiation of RAW 264.7 cells. Proliferating cells express *FoxM1*, which has been shown to play an essential role in cell cycle progression [16,17,18]. However, upon the RES treatment of these differentiating cells, the expression of *FoxM1* increased. This translates in an increment in *FoxM1* expression that will inhibit osteoclast differentiation and increase the proliferation of RAW 264.7 cells. Taken together, our data show the involvement of *FoxM1* on osteoclast differentiation during RES and DOX treatment. 

As FoxM1 is a critical regulator of oxidative responses [45], the upregulation of FoxM1 expression reduces ROS by stimulating the expression of ROS scavenger genes such as *Sod 1* and *Nrf 2* [16,44,45]. During osteoclast differentiation, the expression of *FoxM1* was significantly reduced, suggesting the significant involvement of ROS in this process. RES exposure inhibits osteoclast differentiation and negatively regulates intracellular ROS by stimulating the expression of detoxifying enzymes, such as *Sod 1* and *Nrf 2* [31,32]. In this study, DOX treatment increased osteoclast differentiation and decreased the expression of *Sod 1,* suggesting that *FoxM1* involvement as a regulator of DOX-induced osteoclast differentiation, as previously described by Yao et al. [19] and Olano et al. [20]. Since FoxM1 is a crucial regulator of oxidative stress, DOX and MT [16] both reduced the expression of *FoxM1* in combined treatments, while MT alone or in combination was unable to protect against DOX-induced oxidative stress and osteoclast differentiation. 

In this study, we confirmed the in vitro osteoclast differentiation results through an in vivo experiment using the *ctsk* reporter zebrafish transgenic line (*Tg[ctsk:DsRed*]). DOX treatment significantly increased the *ctsk* positive cells in the head of zebrafish, further confirming our in vitro results on DOX-induced activated osteoclast differentiation on RAW 264.7 cells. On the other hand, exposure to RES decreased the osteoclast differentiation, as shown by *ctsk* positive cells on zebrafish (*Tg[ctsk:DsRed*]). In combination with DOX, RES significantly counteracted the DOX-induced osteoclast differentiation as indicated by *ctsk* positive cells. Apart from osteoclasts, *Ctsk* is present in other tissues such as the ovary, heart, skeletal muscle, lung, placenta, testis, small intestine and colon [57]. The presence of *ctsk* on the intestine has been associated with the pathological conditions of the intestine, e.g., chronic inflammatory disease [58,59]. Similarly to data previously described by Kaczmarek et al. [60], we observed that DOX strongly induced mucositis on our fish model. Interestingly, combining DOX with RES significantly decreased the *ctsk* signal, suggesting that RES could significantly reverse DOX-induced mucositis. The results obtained from the in vivo experiment show that the combination of DOX with RES provides a positive approach that can be implemented in the future to counteract DOX-induced intestinal inflammation. 

Furthermore, DOX exposure in 25 dpf zebrafish post-larvae decreased the locomotor behavior and unsynchronized swimming pattern. DOX-induced locomotor activity might result from impaired sleep–wake rhythms, as described by Savard et al. [61,62]. Similarly, DOX treatment during chemotherapy was associated with disturbances in sleep, sleep efficiency and poor sleep quality [62,63]. The experiments performed with 24 h of exposure to DOX showed unsynchronized swimming behavior as compared to the RES and MT treatments. However, the unsynchronized swimming behavior improved when combining DOX with RES or with MT. These results suggest that RES and MT confer protection to sleep–wake rhythms against DOX-induced stress, although the mechanisms involved are still not fully understood and require further investigation. 

## 4. Materials and Methods

### 4.1. Cells and Cell Culture

RAW 264.7 cells were cultured in Dulbecco’s modified Eagle medium (DMEM; Gibco, Grand Island, NY, USA) supplemented with 10% fetal bovine serum (FBS, Gibco), incubated in a humidified air atmosphere containing 5% CO_2_ at 37 °C. In addition, all culture media were supplemented with 1% penicillin/streptomycin, 2 mM L glutamine and 0.2% fungizone. Penicillin, streptomycin, fungizone and L-glutamine were obtained from Gibco (Gibco). 

### 4.2. XTT Assay

Cytotoxicity was analyzed by XTT assay (Biotium, Fremont, CA, USA). In short, RAW 264.7 cells were seeded in 96-well plates and treated with antioxidants or pro-oxidant. RAW 264.7 cells were treated with RES (TCI, Tokyo, Japan) and MT (Sigma-Aldrich, Darmstadt, Germany) for 4 days, whereas the cells were treated with DOX (TCI) for 3 h. Hereafter, 25 µL of XTT activator reagent was mixed with 5 mL XTT reagent and mixed. Next, 50 µL of freshly prepared XTT reagent (activator + reagent) was added to 100 µL of fresh media 100. Finally, 150 µL of media + XTT was added to each well and incubated for 2–4 h at 37 °C, 5% CO_2_. The absorbance was measured at 630 nm using a microplate reader (Biotek synergy 4, Winooski, VT, USA). Three isolated experiments were performed.

### 4.3. Osteoclast Differentiation

RAW 264.7 cells were differentiated into osteoclasts by induction with M-CSF (30 ng/mL) (Peprotech, London, UK) and RANKL (50 ng/mL) (Peprotech). All differentiation experiments were performed with a control− (growth medium only), a control+ (added cytokines (RANKL and M-CSF) and with treatment using RES (cytokines + RES), MT (cytokines + MT), DOX (cytokines + DOX) and in combinations of antioxidants with DOX (cytokines+ antioxidants + DOX). The cells were exposed to RES or MT continuously for 4 days, whereas exposure to DOX was maintained for 3 h, every 72 h. Treatment media were replaced every 72 h. Three isolated experiments were performed. For in vitro treatments, RES was dissolved in pure ethanol and stored at a concentration of 220 mM, and a working solution was prepared at 10 mM. MT was dissolved and stored at a concentration of 50 mM in distilled water and the working solution was prepared by diluting the stock solution to 1 mM. DOX was dissolved in distilled water and stored at 100 mM, and a working solution was prepared at 100 µM. All antioxidants and pro-oxidant solutions were stored at −20 °C covered with aluminum foil to protect from direct light, and all experiments were conducted at low light conditions.

### 4.4. Tartrate-Resistant Acid Phosphatase Staining (TRAP Staining)

For TRAP staining, RAW 264.7 cells (2.5 × 10^4^ cells/well) were seeded in 24-well plates. Following that, cytokines, M-CSF (30 ng/mL) and RANKL (50 ng/mL) were used for treatment together with antioxidants and pro-oxidant. First, the RAW 264.7 cells were treated with antioxidants (RES and MT) for 4 days whereas the cells were treated with pro-oxidant (DOX) for 3 h. Then, RES, MT, DOX and cytokines were added to the wells, and the medium was renewed after 2 days. Finally, TRAP staining was performed after 4 days of treatment following the Burstone [64] method using Naphthol AS-MX phosphate (Sigma-Aldrich) and Fast Red stain (Sigma-Aldrich). Briefly, the cells are fixed with 4% paraformaldehyde for 10 min. Then, the cells were incubated on buffer containing 50 mM di-sodium tartrate dehydrate on 0.1M acetate buffer, 0.04% MgCl_2_ at pH to 4.4 for 15 min, then the samples were stained with TRAP-staining buffer (incubation buffer + naphthol AS-MX phosphate (0.1 mg/mL) and Fast Red Violet LB (0.3 mg/mL)) in the dark until the TRAP signal was visible. In each well, the number of osteoclastic TRAP positive cells were determined under a microscope and the number of nuclei in each TRAP-positive cell were counted. All the experiments were performed in triplicates. 

### 4.5. RT-PCR and Real-Time PCR

RNA was isolated from the cultures using the Nzyol reagent (Nzytech, Lisbon, Portugal) according to the manufacturer’s instructions. The concentration of RNA was measured by Nanodrop (Thermo Scientific, Waltham, MA, USA). cDNA synthesis was reverse transcribed to cDNA using a kit (Thermo Scientific). 1 µg total RNA from the cells was used for cDNA synthesis. DNA digestion was performed with DNase I treatment (Promega, Madison, WI, USA) for 30 min at 37 °C and reverse-transcribed for 1 h at 37 °C using M-MLV reverse transcriptase (Invitrogen, Waltham, MA, USA), oligo-d(T) universal primer (5′-ACGCGTCGACCTCGAGATCGATG(T)13-3′) and RNaseOUT (Invitrogen). Quantitative real-time PCR (qPCR) assays were performed using the Bio-Rad CFX system (Bio-RAD, Hercules, FL, USA). Gene expression was normalized using β-actin as a housekeeping gene [65], and relative quantification was determined using the ∆∆Ct method [66]. The primers used in this study are listed in Table 1.

### 4.6. Osteoclast Activation In Vivo

To examine osteoclast activation in vivo, a *cathepsin*-K (*ctsk*) reporter zebrafish transgenic line (*Tg[ctsk:DsRed*]) [43] was used. A working solution of RES was prepared at 75 mM and 100 mM in ethanol, MT was prepared at 20 mM and DOX was prepared at 1 mM. Triplicate groups of 15 zebrafish post-larvae at 25 days post-fertilization (dpf) were waterborne exposed to RES (75 µM and 100 µM), MT (20 µM) and DOX (17.2 µM) alone and in combination as follows: DOX + RES 75 (17.2 µM + 75 µM), DOX + RES 100 (17.2 µM + 75 µM) and DOX + MT (17.2 µM + 20 µM) for 24 h. After 24 h of exposure, six post-larvae were randomly selected from each group to analyze the locomotor behavior, wherein each fish was placed in the wells of a 12-well plate for behavior analysis using a Zantiks MWP larvae tracking system (Zantiks Ltd., Cambridge, UK). The post-larvae were acclimated for 15 min in the tracking system, before locomotor behavior was analyzed for 5 min. *Ctsk* signal was identified by fluorescence microscopy using a Leica Mz7.5 stereomicroscope (Leica, Wetzlar, Germany) equipped with an mcherry filter (550–650 nm) and a DFC7000T color camera (Leica). Images were further analyzed using ImageJ1.53c. To measure the fluorescence intensity, the RBG image was split into single channels and converted into grayscale. First, the threshold was adjusted to highlight the fluorescence signal on the head and abdominal region. Then, the intensity was measured, and relative intensity was calculated. 

### 4.7. Statistical Analysis

Statistical analysis was performed using Graphpad prism 8 software (GraphPad Software, Inc., San Diego, CA, USA), and the results are expressed as mean ± SEM. The datasets presenting a normal distribution were evaluated by Student’s *t*-test or one-way analysis of variance (ANOVA) test. Significance of results was considered for *p* ≤ 0.05 (ns—*p* > 0.05, *—*p* ≤ 0.05, **—*p* ≤ 0.01, ***—*p* ≤ 0.001, ****—*p* ≤ 0.0001). All graphics were drawn using GraphPad prism 8. 

## 5. Conclusions

In this study, DOX-induced oxidative stress and osteoclast differentiation are mediated through FoxM1. DOX showed increased osteoclast fusion, differentiation in vitro and in vivo, increased mucositis and changed locomotory behavior and pattern, whereas these phenomena significantly reduced the Sirt1 activator exposure. Furthermore, while combining DOX with the Sirt1 activator (RES), the Sirt1 activator significantly rescued the DOX-induced alteration in osteoclast activation, mucositis and locomotory behavior in vitro and in vivo. Therefore, our observations suggest that the activation of osteoclast differentiation is one of the critical factors for DOX-induced bone loss, where FoxM1 plays a crucial role, and that the Sirt1 activator RES was shown to be able to reverse these negative effects. 

## Figures and Tables

**Table 1:** Primer sequences. All sequences in 5′–3′ orientation.

Gene		Sequences
*Beta-actin*	Forward primer	CCTGACCCTGAAGTACCCCATTGA
	Reverse primer	GTCATCTTTTCACGGTTGGCC
*Oc-stamp*	Forward primer	TGGGCCTCCATATGACCTCGAGTAG
	Reverse primer	TCAAAGGCTTGTAAATTGGAGGAGT
*Rank*	Forward primer	TGCCTCTGGGAACGTGACTG
	Reverse primer	AGGTCTGGCTGACATACACCAC
*Trap*	Forward primer	CAGCTGTCCTGGCTCAAAA
	Reverse primer	ACATAGCCCACACCGTTCTC
*Nfatc1*	Forward primer	CAAGTCCTCACCACAGGGCTCACTA
	Reverse primer	GCGTGAGAGAGGTTCATTCTCCAAGT
*Ctsk*	Forward primer	CTGAAGATGCTTTCCCATATGTGGG
	Reverse primer	GCAGGCGTTGTTCTTATTCCGAG
*Nf-kb p105*	Forward primer	TGTCAACAGATGGCCCATACCT
	Reverse primer	TTGTGACCAACTGAACGATAACCT
*Sod 1*	Forward primer	GGACAATACACAAGGCTGTACCA
	Reverse primer	CAGTCACATTGCCCAGGTCTC
*Nrf 2*	Forward primer	AAAGTTCAGTCTTCACTGCCC
	Reverse primer	TCGGTATTAAGACACTGTAATTCGG
*FoxM1*	Forward primer	GTCTCCTTCTGGACCATTCACC
	Reverse primer	GCTCAGGATTGGGTCGTTTCTG

## Data Availability

Not applicable.
